# Estimating the Proportion of Overdiagnosis among Prostate, Breast, and Thyroid Cancers in China: Findings from the Global Burden of Disease 2019

**DOI:** 10.3390/curroncol31090418

**Published:** 2024-09-20

**Authors:** Shuting Wang, Yanlai Ji, Mingxue Ren, Jun Li, Zuyao Yang

**Affiliations:** 1 JC School of Public Health and Primary Care, Faculty of Medicine, Chinese University of Hong Kong, Shatin, Hong Kong SAR, China; 2 Innovent Biologics, Shanghai 200050, China; 3 Immunoprophylaxis Department, Anhui Provincial Center for Disease Control and Prevention, Hefei 230601, China; 4 Department of Biostatistics and Programming, Sanofi, Chengdu 610000, China

**Keywords:** cancer, lifetime risk, overdiagnosis, Global Burden of Disease

## Abstract

The incidence of prostate, breast, and thyroid cancers has increased in China over the past few decades. Whether and how much these increases can be attributed to overdiagnosis are less understood. This study aimed to estimate the proportion of overdiagnosis among these three cancers in China during 2004–2019. The age-specific cancer incidence, cancer mortality, and all-cause mortality in China were extracted from the Global Burden of Diseases 2019. The lifetime risk of developing and that of dying from each cancer were calculated using the life table method. The proportion of overdiagnosis of a cancer was estimated as the difference between the lifetime risk of developing the cancer and that of suffering from the cancer (including death, metastasis, and symptoms caused by the cancer), further divided by the lifetime risk of developing the cancer. The highest possible values of these parameters were adopted in the estimation so as to obtain the lower bounds of the proportions of overdiagnosis. Sensitivity analyses assuming different lag periods between the diagnosis of a cancer and death from the cancer were performed. The results showed that the lifetime risk of developing prostate, breast, and thyroid cancer increased dramatically from 2004 to 2019 in China, while the increase in the lifetime risk of dying from these cancers was less pronounced. The proportions of overdiagnosis among prostate, breast, and thyroid cancers were estimated to be 7.88%, 18.99%, and 24.92%, respectively, in 2004, and increased to 18.20%, 26.25%, and 29.24%, respectively, in 2019. The increasing trends were statistically significant for all three cancers (all *p* < 0.001). In sensitivity analyses, the proportions of overdiagnosis decreased, but upward trends over time remained for all three cancers. In conclusion, the overdiagnosis of prostate, breast, and thyroid cancers in China increased from 2004 to 2019, with the highest proportion seen in thyroid cancer and the most rapid increase seen in prostate cancer. Multifaceted efforts by policy makers, guideline developers, and clinicians are needed to tackle this problem.

## 1. Introduction

Cancer overdiagnosis has become a global concern in recent years. This happens when cancers that would otherwise not go on to cause symptoms or death are detected [1]. This problem is especially notable in prostate, breast, and thyroid cancer [2,3,4], mainly because these cancers have a reservoir of indolent lesions which are non-progressive or very slowly progressive with a low risk of metastasis and death [1,5]. Such indolent lesions can be identified during screening tests for prostate, breast, and thyroid cancers, which are commonly available nowadays. The early detection of these indolent lesions could lead to overdiagnosis and even overtreatment. 

Over the past several decades, the incidences of prostate, breast, and thyroid cancers in China have increased rapidly [6,7,8], which can be, at least partially, attributable to early cases detected by screening activities. For instance, a previous study from China showed that the proportion of breast cancer detected by screening was increasing across all age groups, with 82% of cases diagnosed at an early stage [9]. However, whether and how much the increase in the incidence of the abovementioned three cancers in China can be attributed to overdiagnosis are not well understood. This study aimed to quantify the potential overdiagnosis of prostate, breast, and thyroid cancers between 2004 and 2019 in China by applying the lifetime conditional probabilities of developing and dying from each cancer, which were calculated based on annual age-specific cancer incidence, cancer mortality, and all-cause mortality, to a hypothetical cohort of 10 million live births. 

## 2. Methods 

### 2.1. Data Source

This study used data from the Global Burden of Diseases 2019, which is a periodical publication of the Global Health Data Exchange supported by the Institute for Health Metrics and Evaluation at the University of Washington [10]. It provides up-to-date data on annual age-specific incidence and mortality for all regions around the world [11]. The detailed protocol of the Global Burden of Diseases database describes methods to retrieve data and is available from the website of the Institute for Health Metrics and Evaluation [12]. Briefly, the cancer data for China were mainly provided by cancer registries spread over the country, which increased from 43 in 2004 to 574 in 2019. The age-specific incidence and mortality of prostate cancer in Chinese men, breast cancer in Chinese women, and thyroid cancer in both Chinese men and women from 2004 to 2019 were extracted for analysis in this study. 

### 2.2. Estimating the Proportion of Cancer Overdiagnosis

In this study, overdiagnosis was defined as the diagnosis of a cancer that would not cause symptoms, metastasis, or death throughout a lifetime. Following this definition, for each year (corresponding to a birth cohort), the lifetime risk of being overdiagnosed with a particular cancer was calculated as the lifetime risk of developing the cancer minus the lifetime risk of developing a cancer that would cause symptoms, metastasis, or death. Let a denote the lifetime risk of developing the cancer; b denote the lifetime risk of dying from the cancer (fatal cancer); c denote the lifetime risk of developing a metastatic, non-fatal cancer; and d denote the lifetime risk of developing a symptomatic, non-metastatic, non-fatal cancer. Then, the lifetime risk of being overdiagnosed with cancer is equal to a − b − c − d. Of note, b included the observed mortality from a cancer (b1) and the mortality prevented by cancer screening and treatment (b2, which was unobservable), and b2 was estimated to be up to 20% of b, according to previous randomized controlled trials that evaluated the effectiveness of cancer screening programs [13,14]. Consequently, b was equal to b1 × 1.25. The calculation of a and b1 is illustrated in detail in the Statistical Analyses Section and the Supplementary Methods. The third parameter, c, was estimated to be up to 20% of b1, according to a previous study [1]. The fourth parameter, d, was estimated to be up to 50% of (a − b − c), according to the proportion of symptomatic cancers found at the time of diagnosis [15,16,17]. This value of 50% was an overestimated number, because it assumed that all cancer patients who were symptomatic at diagnosis would not develop metastasis or die from cancer. With the above assumptions, the lifetime risk of being overdiagnosed can be rewritten as follows: a − b1 × 1.25 − b1 × 20% − (a − b1 × 1.25 − b1 × 20%) × 50% = 0.5 × a − 0.725 × b1. This estimated risk should be regarded as the lower bound because, as mentioned above, the highest possible values were used for b2, c, and d. After we calculated this estimated risk, the proportion of overdiagnosis among the diagnosed cancers was calculated by dividing the estimated risk by a, i.e., (0.5 × a − 0.725 × b1)/a. 

### 2.3. Statistical Analyses

a and b1 in the above equation were calculated using the life table method based on cancer incidence and mortality data from the same year. This method is widely recognized as a reliable statistical method and has been extensively used in cancer agencies worldwide to calculate the lifetime risks of being diagnosed with cancer and of dying from cancer [18,19]. Firstly, a hypothetical cohort of 10 million live births was constructed. The cohort was stratified into 18 age intervals in accordance with the Global Burden of Diseases 2019: <1, 1 to 4, 5 to 9, 10 to 14, 15 to 19, 20 to 24, 25 to 29, 30 to 34, 35 to 39, 40 to 44, 45 to 49, 50 to 54, 55 to 59, 60 to 64, 65 to 69, 70 to 74, 75 to 79, and 80 years old. The age-specific incidence and mortality of prostate, breast, and thyroid cancer extracted from the Global Burden of Diseases 2019 were applied to this cohort to estimate the number of cancer cases, cancer deaths, survivors, and cancer-free survivors (i.e., people who were alive and free of a specific cancer) in the life table. Then, a was calculated by dividing the total number of new cancer cases across all age intervals by 10 million, and b1 was calculated by dividing the total number of cancer deaths across all age intervals by 10 million. This method assumed that (1) there was no change in the underlying risk factors for prostate, breast, and thyroid cancer over time, and (2) subjects who died from other causes had not developed the cancer of interest. The details of the calculations can be found in the Supplementary Methods and Supplementary Tables. The trend in the proportion of overdiagnosis over years was tested using a linear regression model for each cancer.

### 2.4. Sensitivity Analyses

In the main analyses, the incidence and mortality data were from the same year. However, people who are diagnosed with a cancer in a certain year may not die from it in the same year but instead years later (i.e., there is a lag). In other words, the risk of cancer mortality calculated based on data from the same year may not represent the risk of cancer mortality of the people who were diagnosed that year. To examine the impact of this issue on the main results, we performed two sensitivity analyses which assumed that people diagnosed with cancer in a certain year died 5 years later and 10 years later. The 5-year and 10-year lags were assumed based on the time from diagnosis to death among patients who eventually died from these three cancers. The median time to cancer death is 5 years for breast cancer, 5 years for prostate cancer, and 2 years for thyroid cancer, respectively [20,21,22]. More than 70% of deaths caused by these three cancers occur within 10 years of diagnosis [20,21,22]. Thus, for simplicity, the 5-year and 10-year lags were applied to all three cancers. In the sensitivity analyses, the four parameters in the equation were estimated based on the mortality data from 5 or 10 years later. For example, for the year 2004, the four parameters were calculated based on the mortality data from 2009 (assuming a 5-year lag) or 2014 (assuming a 10-year lag). Since the latest mortality data used in this study were from the year 2019, when assuming a 5-year lag, the lifetime risk of being overdiagnosed was re-estimated for the years 2004–2014 (i.e., 5 years prior to 2019) only, and when assuming a 10-year lag, the lifetime risk of being overdiagnosed was re-estimated for the years 2004–2009 (i.e., 10 years prior to 2019) only. For the reason explained above, an additional 2-year lag was assumed for thyroid cancer, and in the corresponding sensitivity analysis, the lifetime risk of being overdiagnosed with thyroid cancer was re-estimated for the years 2004–2017 (i.e., 2 years prior to 2019). 

The statistical analyses and plots were performed and created using R software (version 4.3.1), and the values were calculated using Microsoft Excel (https://www.microsoft.com/en-us/microsoft-365/excel, accessed on 18 September 2024) spreadsheets in this study. For the trend test, a two-sided *p* value < 0.05 was considered statistically significant. 

## 3. Results

The estimations of the lifetime risk of developing prostate, breast, and thyroid cancers and that of dying from the cancers are shown in Table 1. From 2004 to 2019, the lifetime risk of developing prostate, breast, or thyroid cancer increased rapidly by 75% (from 1.315% to 2.307%), 59% (from 2.595% to 4.133%), and 60% (from 0.133% to 0.213%), respectively. The lifetime risk of dying from cancer also increased during this period, but the change was less pronounced: 32% (from 0.764% to 1.012%) for prostate cancer, 22% (from 1.110% to 1.354%) for breast cancer, and 33% (from 0.046% to 0.061%) for thyroid cancer. As estimated on the basis of contemporaneous incidence and mortality (i.e., data from the same year), the proportions of overdiagnosis among prostate, breast, and thyroid cancers were 7.88%, 18.99%, and 24.92%, respectively, in 2004, and steadily increased to 18.20%, 26.25%, and 29.24%, respectively, in 2019 (Table 1). The increasing trends were statistically significant for all three cancers (all *p* < 0.001). The highest proportion of overdiagnosis was seen in thyroid cancer across all years, while the most rapid increase in proportion was seen in prostate cancer (more than doubled in 2019 compared to 2004, Figure 1).

In the sensitivity analyses, assuming a 5-year lag or a 10-year lag between the diagnosis of a cancer and death from cancer, the proportion of overdiagnosis decreased, but the upward trend over time remained. When a 5-year lag was assumed, the proportions of overdiagnosis among prostate, breast, and thyroid cancers were 7.46%, 18.68%, and 21.52%, respectively, in 2004, and steadily increased to 14.90%, 23.09%, and 27.74%, respectively, in 2014 (Table 2). When a 10-year lag was assumed, the proportions of overdiagnosis among prostate, breast, and thyroid cancers were 5.96%, 16.92%, and 18.91%, respectively, in 2004, and increased to 10.55%, 19.82%, and 25.25%, respectively, in 2009 (Table 3). When an additional 2-year lag was assumed for thyroid cancer, the proportion of overdiagnosis was 23.75% in 2004 and steadily increased to 28.21% in 2017. 

## 4. Discussion

Overdiagnosis can cause significant harm to patients, both physically (e.g., removal of organs by major surgery, adverse effects of chemotherapy) and psychologically (e.g., anxiety and distress), and place heavy burden on the healthcare system. This study showed that the proportion of overdiagnosis among prostate, breast, and thyroid cancers in China increased steadily from 2004 to 2019, with thyroid cancer being the most affected and prostate cancer being the fastest-growing (more than doubled). 

This increased overdiagnosis could be partly explained by the urbanization, increased social medical insurance coverage, and increased health awareness of people in China. Urbanization improves the accessibility of healthcare as more people have the chance to be examined in hospitals. Additionally, social medical insurance coverage in China increased from 29.7% in 2003 to 95.7% in 2011 [23], and cancer screening, such as ultrasound scans for breast and thyroid cancer, is covered by basic medical insurance for urban employees in many health examination centers in China. This has encouraged frequent access to health checks and screening which may increase the chance of detecting indolent cancers. The health awareness of the Chinese population has also been improving over the past several decades. However, due to the lack of evidence-based guidelines regarding screening and the fact that the general public has no capacity to make evidence-based decisions, the enthusiasm in the pursuit of health often results in unnecessary health checks and treatments.

To the best of our knowledge, no previous studies have investigated the magnitude of the overdiagnosis of prostate and breast cancers in China. A previous study by Li et al. estimated that the overdiagnosis of thyroid cancer was over 70% in urban areas and 60% in rural areas in China [24], which were much higher than our estimates. This difference could be partly attributed to the different methodology they adopted. Specifically, in the study of Li et al., the proportion of overdiagnosis was estimated as the difference between the observed and expected age-specific incidences, assuming that the latter were the same as those observed in countries with long-standing registries, such as the Nordic countries, before the 1970s. However, it should be noted that the incidence age curve usually varies across different populations because of the difference in their exposure to risk factors of thyroid cancer. A number of studies from other countries have estimated the magnitude of cancer overdiagnosis but yielded heterogeneous results, with the proportions of overdiagnosis ranging from 1.7% to 67% for prostate cancer [2], from 0% to 57% for breast cancer [25], and from 60 to 90% for thyroid cancer [26]. These estimates are hardly comparable, as they varied greatly across different populations, screening protocols, and methods used [2]. 

In this study, the proportion of overdiagnosis was estimated based on the differences between the lifetime risk of developing cancer and that of suffering from cancer, including cancer deaths, cancer metastasis, and cancer symptoms. This approach aligned with the definition of overdiagnosis given by the US National Library of Medicine, i.e., “the labeling of a person with a disease or abnormal condition that would not have caused the person harm if left undiscovered” [27]. Previous studies that estimated the proportion of overdiagnosis were also based on the three components, namely the symptoms, metastasis, and deaths caused by cancers [1,25,28]. Thus, we believe the definition of overdiagnosis used in this study is valid and would not cause significant impacts on our findings. We did not use the excess lifetime risk method, which has been widely used in other studies [29,30], because its assumption that screening is well established or organized in the study population is not satisfied in our study setting. Cancer screening activities in China are mainly opportunistic [31,32,33]. Although two rounds of organized breast cancer screening programs have been carried out since 2009, only a limited number of women attended these screening [34,35,36]. The approach we adopted allowed for the estimation of all types of overdiagnosis, not only limited to overdiagnoses caused by organized screening programs. 

Our findings suggest that the overdiagnosis of cancer in China is increasing. Multifaceted efforts are needed to tackle this problem. Policy makers such as the authorities regulating medical insurance may adjust reimbursement policies to reduce the uptake of screening examinations that are not supported by evidence. Guideline developers should endeavor to follow the “guidelines for developing practice guidelines” [37] and ensure that the guidelines developed are based on the currently available best evidence and also carefully consider the benefits and harms before recommending any cancer screening. Unfortunately, this is often not the case in reality, particularly in China. For example, no randomized trials have ever evaluated the effectiveness of thyroid cancer screening in reducing mortality, and existing trials of prostate cancer screening have shown that it does not reduce cancer-specific or all-cause mortality, but screening for these two cancers is still offered during routine health check-ups and remains unregulated in China [33,38]. In the presence of early-stage or micro cancers that are diagnosed, it is important for clinicians to inform patients of the benefits and risks of an aggressive strategy (e.g., immediate surgery) versus a conservative strategy (e.g., watchful waiting) for management, taking patients’ value into account, so as to minimize potential overtreatment.

Our study has some limitations. Firstly, the life table method using annual data to calculate the proportion of cancer overdiagnosis assumes that there are no temporal changes in the underlying risk factors for breast, prostate, and thyroid cancer, which may not exactly reflect real-world situations. However, the increasing trend of overdiagnosis remained even when 5-year and 10-year lags between the diagnosis of and death from cancer were applied, suggesting that our main finding was not affected by this issue. Secondly, when estimating the percentage of metastatic and other symptomatic cancers relative to mortality, the parameters we used were based on previous studies and were subject to uncertainty. For this reason, we had to use the highest possible values to obtain conservative estimates. In other words, the proportions of overdiagnosis reported in this study represent the lower bounds of the actual proportions, indicating that the magnitude of overdiagnosis in China could be even more significant. Thirdly, we were not able to examine the impact of longer lags (e.g., 15 years and 20 years) between cancer diagnosis and cancer death in the sensitivity analysis, because the data used in this study spanned only 15 years (from 2004 to 2019). Fourthly, the accuracy and reliability of China’s cancer registry data in the early years (before 2008) were not satisfactory due to the limited coverage of cancer registration [39,40,41], although no unusual patterns of incidence or mortality were observed in those early years.

In conclusion, our findings suggest that the overdiagnosis of prostate, breast, and thyroid cancers in China has been increasing rapidly, with the highest proportion seen in thyroid cancer and the most rapid increase seen in prostate cancer. Multifaceted efforts by policy makers, guideline developers, and clinicians are needed to tackle this problem.

## Figures and Tables

**Figure 1:**
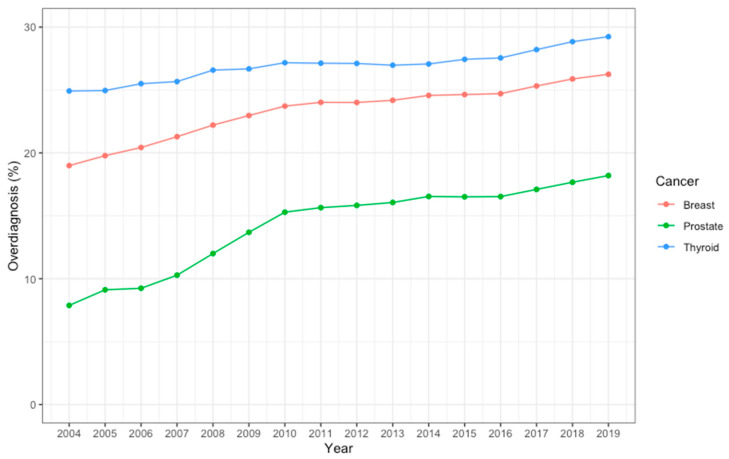
The proportion of overdiagnosis among prostate, breast, and thyroid cancer from 2004 to 2019.

**Table 1:** The lifetime risk and proportion of overdiagnosis among prostate, breast, and thyroid cancer from 2004 to 2019.

Estimate (%)	Cancer	2004	2005	2006	2007	2008	2009	2010	2011	2012	2013	2014	2015	2016	2017	2018	2019
Lifetime risk of developing cancer (a) ^#^	Prostate	1.315	1.378	1.448	1.526	1.591	1.679	1.763	1.815	1.863	1.912	2.000	2.048	2.073	2.177	2.238	2.307
	Breast	2.595	2.680	2.758	2.879	2.995	3.128	3.275	3.360	3.400	3.459	3.604	3.645	3.669	3.866	4.036	4.133
	Thyroid	0.133	0.139	0.145	0.152	0.161	0.171	0.181	0.187	0.190	0.192	0.196	0.196	0.197	0.203	0.209	0.213
Lifetime risk of dying from cancer (b1) ^#^	Prostate	0.764	0.777	0.814	0.836	0.834	0.841	0.844	0.860	0.878	0.895	0.923	0.946	0.957	0.988	0.998	1.012
	Breast	1.110	1.117	1.125	1.140	1.148	1.166	1.187	1.204	1.219	1.232	1.264	1.275	1.280	1.316	1.343	1.354
	Thyroid	0.046	0.048	0.049	0.051	0.052	0.055	0.057	0.059	0.060	0.061	0.062	0.061	0.061	0.061	0.061	0.061
Proportion of overdiagnosis (=(0.5 × a − 0.725 × b1)/a)	Prostate	7.88	9.12	9.24	10.28	12.00	13.69	15.29	15.65	15.83	16.06	16.54	16.51	16.53	17.10	17.67	18.20
	Breast	18.99	19.78	20.43	21.29	22.21	22.97	23.72	24.02	24.01	24.18	24.57	24.64	24.71	25.32	25.88	26.25
	Thyroid	24.92	24.96	25.50	25.67	26.58	26.68	27.17	27.13	27.11	26.97	27.07	27.44	27.55	28.21	28.84	29.24

^#^ The lifetime risk of developing and dying from cancer was rounded to 3 decimal places because the variations across different years were very small.

**Table 2:** The proportion of overdiagnosis among prostate, breast, and thyroid cancer from 2004 to 2014 assuming a 5-year lag between the diagnosis of and death from cancer.

Proportion (%)	Cancer	2004	2005	2006	2007	2008	2009	2010	2011	2012	2013	2014
Overdiagnosis	Prostate	7.46	8.85	9.27	10.14	11.49	12.36	13.43	13.93	14.02	14.09	14.90
	Breast	18.68	19.02	19.31	20.18	21.06	21.47	22.49	23.00	22.45	22.27	23.09
	Thyroid	21.52	21.56	21.62	22.12	23.34	24.71	26.10	26.90	27.19	27.36	27.74

**Table 3:** The proportion of overdiagnosis among prostate, breast, and thyroid cancer from 2004 to 2009 assuming a 10-year lag between the diagnosis of and death from cancer.

Proportion (%)	Cancer	2004	2005	2006	2007	2008	2009
Overdiagnosis	Prostate	5.96	6.68	7.25	8.07	9.27	10.55
	Breast	16.92	17.53	18.08	18.37	18.91	19.81
	Thyroid	18.91	20.07	21.14	22.39	23.84	25.25

## Data Availability

The data used in this study are available on the Global Burden of Disease website at https://www.healthdata.org/research-analysis/gbd (accessed on 18 November 2022).

## Supplementary Materials

The following supporting information can be downloaded at https://www.mdpi.com/article/10.3390/curroncol31090418/s1.
