# Supplementary material for: Estimating the Proportion of Overdiagnosis among Prostate, Breast, and Thyroid Cancers in China: Findings from the Global Burden of Disease 2019
Source: Curr Oncol. 2024 Sep 20;31(9):5643–51. doi: 10.3390/curroncol31090418 (PMC11431510; doi:10.3390/curroncol31090418)
Supplement: Supplementary file 1 [file curroncol-31-00418-s001.zip › curroncol-3137560-Supplementary Materials.pdf]

Supplementary Materials

Supplementary Methods

Supplementary Table S1. The cumulative probability of dying of thyroid cancer in China in 2019

Supplementary Table S2. The cumulative probability of developing thyroid cancer in China in 2019

Supplementary Methods

Calculating lifetime risk of dying from cancer (b_1)

The probability of dying in x to x+n age interval (q_x) was firstly calculated using age-specific all-cause mortality (m_x), with the equation $q_x = \frac{(n \times m_x)}{(1+n/2 \times m_x)}$. The probability of dying in the last age interval (≥80) was assumed to be 1. The number of deaths in x to x+n age interval (d_x) was calculated by q_x multiplied by the number of subjects surviving to age x (l_x, l_x in the first age group was 10 million), with the equation $d_x = q_x \times l_x$. Then the number of subjects surviving to age x+n (l_{x+n}) equaled to the number of survivors by age x minus the number of deaths during the age interval x to x+n ($l_{x+n} = l_x - d_x$). The number of deaths due to a specific cancer in age interval x to x+n (d_x^c) was calculated by d_x multiplied by the proportion of cancer death rate in the age interval x to x + n, with the equation $d_x^c = d_x \times m_x^c/m_x$. The lifetime risk of dying from cancer (b_1) was the sum of d_x^c in each age group and divided by 10 million ($b_1 = \sum_{i=n}^{n} d_x^c/10^7$). An example of the calculation of the lifetime risk of dying from thyroid cancer in 2019 is shown in Supplementary Table 1.

Calculating lifetime risk of developing cancer (a)

The number of people who were alive and free of a specific cancer in the first age interval (<1 year) was assumed to be 10 million. During the age interval "x to x+n", the number of people who were alive and free of a specific cancer by the age of x+n (K_{x+n}) equaled to the number of people who were alive and free of a specific cancer at the beginning of this age interval (K_x) minus the sum of the number of new cancer cases developed during this age interval (C_x) and the number of deaths due to other causes during this age interval (d_x', $d_x' = d_x - d_x^c$), that was $K_{x+n} = K_x - C_x - d_x'$. Then the probability of developing a specific cancer in age group x to x + n (p_x) is calculated with the equation $p_x = \frac{I_x \times (1-(1-I_x)^n)}{I_x}$. It represents the possibility of people who are

alive and free of a specific cancer at x-year-old developing this specific cancer in the next n years. The number of new cancer cases in age interval x to x + n (C_x) was then calculated with the equation $C_x = K_x * p_x$. Finally, the lifetime risk of developing a specific cancer (a) was equal to the sum of new cancer cases in each age interval and divided by 10 million ($a = \sum_{i=n}^{n} C_x^c / 10^7$). An example of the calculation of the lifetime risk of developing thyroid cancer in 2019 is shown in Supplementary Table 2.

Supplementary Table S1. The cumulative probability of dying of thyroid cancer in China in 2019

Age	All-cause mortality (**m_x**, $1/10^5$**)**	The probability of dying **(q_x)**	No. of alive **(l_x)**	No. all-cause death **(d_x)**	Thyroid cancer mortality **(m_x^c**, $1/10^5$**)**	No. that died of thyroid cancer **(d_x^c)**	Cumulative probability of dying of thyroid cancer (%)
<1 year	676.54	0.006743	10000000	67426	0.00	0	0.000
1 to 4	43.30	0.001731	9932574	17189	0.00	0	0.000
5 to 9	25.81	0.001290	9915385	12787	0.01	7	0.000
10 to 14	21.23	0.001061	9902598	10505	0.02	8	0.000
15 to 19	35.88	0.001792	9892094	17730	0.02	8	0.000
20 to 24	55.74	0.002783	9874364	27482	0.03	15	0.000
25 to 29	60.22	0.003006	9846882	29604	0.04	22	0.001
30 to 34	84.11	0.004197	9817278	41200	0.06	29	0.001
35 to 39	123.48	0.006155	9776078	60173	0.09	45	0.001
40 to 44	186.48	0.009281	9715906	90170	0.18	85	0.002
45 to 49	247.68	0.012308	9625736	118470	0.25	118	0.003
50 to 54	393.64	0.019490	9507266	185298	0.45	213	0.006
55 to 59	616.80	0.030372	9321968	283123	0.74	339	0.009
60 to 64	996.77	0.048627	9038845	439528	0.75	330	0.012
65 to 69	1617.44	0.077729	8599317	668414	1.12	464	0.017
70 to 74	2950.20	0.137378	7930903	1089529	1.70	627	0.023
75 to 79	4973.94	0.221192	6841374	1513257	4.10	1247	0.036
>80	12329.41	1.000000	5328117	5328117	5.95	2570	**0.061**

Supplementary Table S2. The cumulative probability of developing thyroid cancer in China in 2019

Age	Thyroid cancer incidence (**I_x**, 1/10^5)	The probability of developing thyroid cancer (**p_x**)	Total No. alive and free of thyroid cancer (**K_x**)	No. that died of other causes (**d_x'**)	No. that developed thyroid cancer (**C_x**)	Cumulative probability of developing thyroid cancer (%)
<1 year	0.00	0	10000000	67426	0	0.000
1 to 4	0.00	0	9932574	17189	0	0.000
5 to 9	0.29	0.000014	9915385	12779	144	0.001
10 to 14	0.27	0.000014	9902462	10497	136	0.003
15 to 19	0.35	0.000018	9891829	17722	175	0.005
20 to 24	0.60	0.000030	9873932	27467	297	0.008
25 to 29	1.00	0.000050	9846169	29582	494	0.012
30 to 34	1.90	0.000095	9816093	41171	933	0.022
35 to 39	2.56	0.000128	9773989	60128	1250	0.034
40 to 44	4.13	0.000207	9712612	90085	2008	0.054
45 to 49	3.44	0.000172	9620519	118351	1655	0.071
50 to 54	4.63	0.000232	9500512	185085	2201	0.093
55 to 59	5.37	0.000269	9313226	282783	2502	0.118
60 to 64	4.39	0.000219	9027941	439199	1981	0.138
65 to 69	4.30	0.000215	8586762	667950	1848	0.156
70 to 74	4.38	0.000219	7916965	1088902	1735	0.174
75 to 79	6.67	0.000333	6826328	1512011	2275	0.196
>80	6.39	0.000319	5312042	5325547	1697	**0.213**
